# In Vivo and In Vitro Antioxidant Activities of Methanol Extracts from Olive Leaves on *Caenorhabditis elegans*

**DOI:** 10.3390/molecules24040704

**Published:** 2019-02-15

**Authors:** Siyuan Luo, Xuelian Jiang, Liping Jia, Chengyue Tan, Min Li, Qiuyu Yang, Yanlin Du, Chunbang Ding

**Affiliations:** College of Life Science, Sichuan Agricultural University, Ya’an 625014, China; luosiyuan1998@163.com (S.L.); 15397753946@163.com (X.J.); 15983630065@163.com (L.J.); tanchengyue7@163.com (C.T.); limin18398262832@163.com (M.L.); yangqiuyu145@163.com (Q.Y.); 18123427338@163.com (Y.D.)

**Keywords:** olive leaves, polyphenols, antioxidant activities, *Caenorhabditis elegans*, thermal stress

## Abstract

The aim of this study was to evaluate the antioxidant activities of extracts from olive leaves (EOL). The main contents of EOL were determined by colorimetric methods. The antioxidant activities were assessed by measuring the scavenging free radicals in vitro. To investigate the antioxidant activity in vivo, we detected the survival of *Caenorhabditis elegans*, under thermal stress. Subsequently the reactive oxygen species (ROS) level, activities of antioxidant enzymes, the expression of HSP-16.2 and the translocation of *daf-16* were measured. The results showed that, polyphenols was the main component. EOL could well scavenge DPPH and superoxide anion radicals in vitro. Compared to the control group, the survival rate of *C. elegans* treated with EOL was extended by 10.43%, under heat stress. The ROS level was reduced, while the expression of *hsp-16.2* was increased to protect the organism against the increasing ROS. The level of malondialdehyde (MDA) also decreased sharply. The activities of inner antioxidant enzymes, such as catalase (CAT), superoxide dismutase (SOD), and glutathione peroxidase (GSH-PX) were potentiated, which might have had a correlation with the DAF-16 transcription factor that was induced-turned into the nuclear. Therefore, EOL showed a strong antioxidant ability in vitro and in vivo. Hence, it could be a potential candidate when it came to medicinal and edible plants.

## 1. Introduction

Olive (*Olea europare* L.) deriving from the Mediterranean was introduced to China in the 1960s. The oil from olive fruits had been historically used as a kind of cooking oil in the Mediterranean. Additionally, olive oil had rich monounsaturated fatty acids and a wide array of bioactive compounds, such as polyphenols, flavonoids, and squalence, which were also renowned as liquid gold [1]. Studies have showed that olive oil has a wide range of pharmacological applications, such as reduction in blood pressure and anti-bacterial, anti-aging, and anti-tumor activity; these effects are more prominently seen is Europe [2,3,4]. Olive fruit extracts are also widely used as nutrition supplements, food additives, and medicinal and ceremonial ingredients. Consequently, olive has attracted the attention of the world, due to its highly beneficial effect on humans [5]. By 2012, the cultivation area of olive trees, in China, had reached over three million hectares. According to statistics, approximately 25 kg olive leaves are produced from one olive tree, every year, after the pruning of branches. The majorities of olive leaves are abandoned or incinerated, which leads to environmental pollution and resource waste [6]. Studies have shown that the main compounds in olive leaves are the total polyphenol and the total flavonoids [7]. The total polyphenol content in olive leaves is more than that in olive fruits [8]. As is known, polyphenol from plants shows a strong antioxidant activity [9]. The polyphenols can remove the over-expression of free radicals in cells, reduce oxidative damage, and reduce inflammation, aging, or neurodegenerative diseases induced by reactive oxygen species [10]. However, the utilization rate of olive leaves is lower, compared to that of olive fruits. So olive leaves can be an abundant source of polyphenol content.

During the cell metabolism process, a series of reactive oxygen species (ROS) are produced, such as superoxide anion, hydroxyl radicals, and hydrogen peroxide [11]. The redundant free radicals can damage cells by destructing protein to accelerate the development of aging, inflammation, and lipid peroxidation [12,13]. Additionally, high concentrations of ROS can induce cell apoptosis [14]. Recent evidence has shown that olive oil can scavenge free radicals [15]. Marino et al. found the crude olive leaves extracts could also significantly scavenge the DPPH free radical, and EC50 was 62.6 µM [16].

*Caenorhabditis elegans* can be easily cultivated in the laboratory as they feed on *Escherichia coli*. Their life-cycle from the fertilized eggs to adult worms is only 3.5 days. The whole body of the *C. elegans* is transparent, making it easy to observe by a microscope, without using a dye. Most importantly, its complete genomic sequencing has already been conducted. Therefore, *C. elegans* as a model organism is widely applied for screening bioactive compounds [17]. There is a tight connection between lifespan extension and resistance to diverse environments [18]. Liao et al. found that the curcumin could prolong the lifespan, while showing a greater survival rate in thermal and oxidative stress [19]. Extensive reports have demonstrated that plants extracts can enhance resistance in *C. elegans*, under acute stress [20,21]. Antioxidant enzymes like catalase (CAT), superoxide dismutase (SOD), glutathione peroxidase (GSH-P_X_) are the main defense system to protect organisms from increasing ROS. Some studies have reported that when pretreated with extracts of plants, inner antioxidant enzymes are promoted under oxidative stress. Ishikado et al. have also found that after pretreatment with willow bark extracts, the activities of inner antioxidative enzymes are promoted under oxidative stress [22]. Recent research work has reported that polyphenol compounds could prolong lifespan and raise resistance in *C. elegans*, under oxidative stress [23]. However, reports on extracts from olive leaves acting on *C. elegans*, are rare.

In this study, the main components of methanol extracts from olive leaves (EOL) were determined by the colorimetric method. The antioxidant capacity of EOL was evaluated in vitro, by determining its reducing power, DPPH, and superoxide anion scavenging abilities. Using *C. elegans* as the model organism, the antioxidant capabilities of EOL was evaluated in vivo, by detecting the survival rate, ROS level, and antioxidant enzyme activities of *C. elegans*, under thermal stress. Moreover, we used transgenic *C. elegans* strains to investigate the antioxidant mechanism. The results will provide theoretical guidance on the utilization and development of olive leaves.

## 2. Results and Discussion

### 2.1. Components of EOL

As showed in Table 1, polyphenols and flavonoids were the main contents of EOL, accounting for 41.77 ± 2.38% and 30.01 ± 0.76%, respectively. A similar outcome was obtained by Le et al., who showed extracts obtained from olive leaves contained about 45% polyphenol compounds [24]. The content of proteins in this study was found to reach to 20.40 ± 0.29%. However, free amino acids was only about 0.09 ± 0.02% and soluble sugars was only 14.14 ± 0.29%.

### 2.2. In Vitro Antioxidant Activities

A single antioxidant assay could not evaluate the antioxidant capacity of extracts, comprehensively. Several testing methods should be carried out to value the antioxidant abilities of plant extracts [25,26]. Therefore, in this study, the antioxidant activity of EOL was estimated by determining its scavenging effect on DPPH, superoxide anion radicals, and its reducing power.

**Figure 1:**
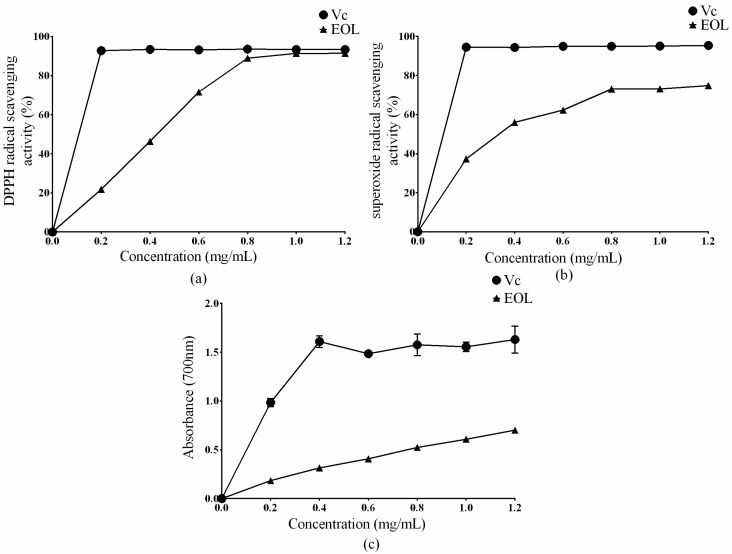
Effect of EOL on free radicals in vitro. (**a**) The DPPH radical scavenging effect, (**b**) the superoxide radical scavenging effect, and (**c**) the reducing power.

DPPH is widely used as a tool to estimate the free radical scavenging activities of antioxidants. As shown in Figure 1a, the maximum scavenging ability of EOL on the DPPH radical was 91.03%, at 1.2 mg/mL. After 0.8 mg/mL, there was no significant difference between the DPPH scavenging ability of the EOL and ascorbic acid (Vc) (*p* > 0.05). The result was in agreement with previous studies. Brahmi et al. found that olive leaves extracts could well scavenge DPPH, by 90% [27]. The IC_50_ of EOL was found to be 0.38 mg/mL (IC_50_ refers to the concentrations at which 50% of the DPPH radical was scavenged). The result was well in accordance with the research that the DPPH IC_50_ values of olive leaves extracts varied from 0.17 to 0.97 mg/mL [28].

As shown in Figure 1b, the EOL could scavenge the superoxide anion free radical, with a maximum clearance of 73.82% at 1.2 mg/mL and an IC_50_ = 0.33 mg/mL. Compared with Vc, the absorbance of EOL was comparatively low, but the tendency of the curve ascended, meaning the reducing power of EOL conformed to the dose-dependent effect (Figure 1c).

A high reducing activity at a lower concentration indicated a potential higher antioxidant ability. Studies have demonstrated that the reducing power of bioactive compounds is related to its antioxidant activity [29,30]. Other experiments have showed that a higher content of the total polyphenol and total flavonoid could contribute to a higher biological activity. Falleh found that the polyphenol from *Cynara cardunculus* L. leaves displayed a good scavenging radical ability [31]. Xian et al. also discovered that the flavonoid extracts from Ferns could scavenge about 95% of the superoxide anion radical [32]. In this study, polyphenols were the main compounds in EOL (Table 1). Therefore, the polyphenols and the flavonoids could be the main reason for a high antioxidant activity in vitro. Antioxidant activity assays in vitro were consistent with previous work [33].

### 2.3. In Vivo Biological Activities

#### 2.3.1. Determination of a Suitable Concentration

**Figure 2:**
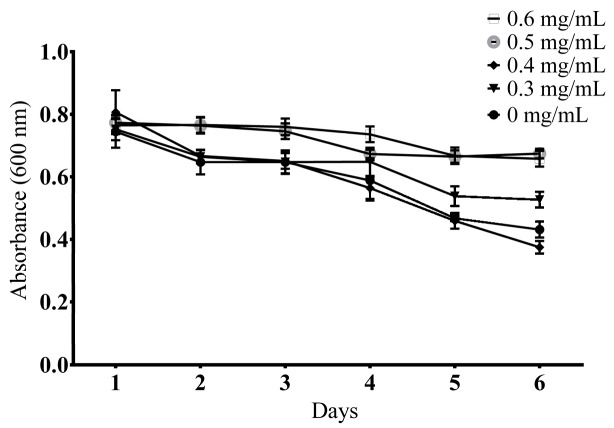
The daily OD values of *Escherichia coli* OP50 for each concentration level of EOL.

Worms could reproduce a number of offspring that quickly consumed the limited OP50 supply. Therefore, the OD value of the wells without EOL, significantly descended (Figure 2). The addition of 0.4 mg/mL to the mediums, showed no notable effect on food clearance, compared to the control, while worms treated with 0.3 mg/mL, 0.5 mg/mL, and 0.6 mg/mL had a negative effect on food clearance. Based on the above analysis, 0.4 mg/mL was found to be the most suitable concentration.

#### 2.3.2. Verification of the Non-Toxic Concentration

**Figure 3:**
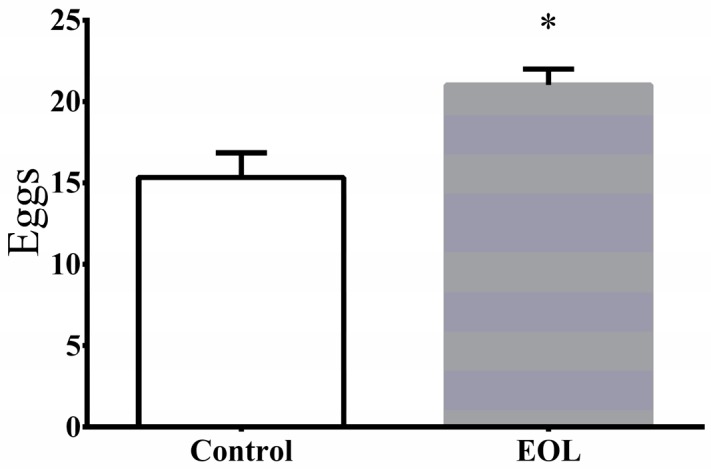
The eggs laid by the *C. elegans*. Differences were considered significant at *p* < 0.05 (*).

To find out the suitable concentration of treatment for worms, the fertilization in *C. elegans* was investigated. After treatment by EOL, for 48 h, the experimental group (laid 21 ± 1) showed a 36.96% increase in progeny, compared to the control group (laid 15.33 ± 1.53) (Figure 3). The result did not indicate that this level of concentration had a toxic effect on *C. elegans*. Therefore, this concentration level was considered to be suitable for the following experiment.

#### 2.3.3. Survival under Thermal Stress

**Figure 4:**
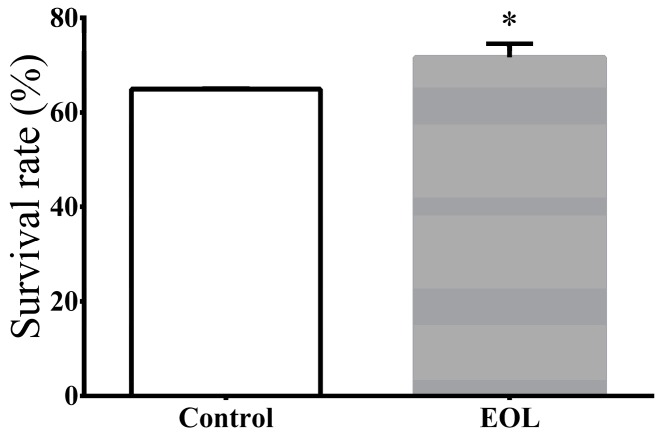
The survival rate of *C. elegans* under thermal stress. Differences were considered significant at *p* < 0.05 (*).

Extracts from plants could improve the resistance in *C. elegans* under thermal stress. Extra virgin olive oil could extend the survivals of worms, under heat stress [23]. Vayndorf and co-workers found extracts from apple could strength resistance to heat, UV radiation, and oxidative stress [34]. The polyphenol from the Zalema grape pomace could also increase the resistance in *C. elegans*, under thermal stress [35]. Hence to investigate the effect of EOL on heat resistance in *C. elegans*, treated worms were exposed to 35 °C, for 5 h. The result showed that the EOL heightened the resistibility of *C. elegans*, by 10.43% (Figure 4).

Plant extracts of acai palm, apple, asparagus, blueberry, cinnamon, and cocoa, have been proved to have active antioxidant defense mechanisms to increase survival rates of organisms under environmental stress [36]. The thermal stress resistance effect of polysaccharides from *Panax notoginseng* on *C. elegans* was augmented, as it raised the antioxidant enzyme activities and sharply decreased the ROS levels [37,38]. In this study, we assessed the ROS levels to make a further exploration of how EOL increases the resistance in *C. elegans* under acute stress.

#### 2.3.4. The ROS Level

**Figure 5:**
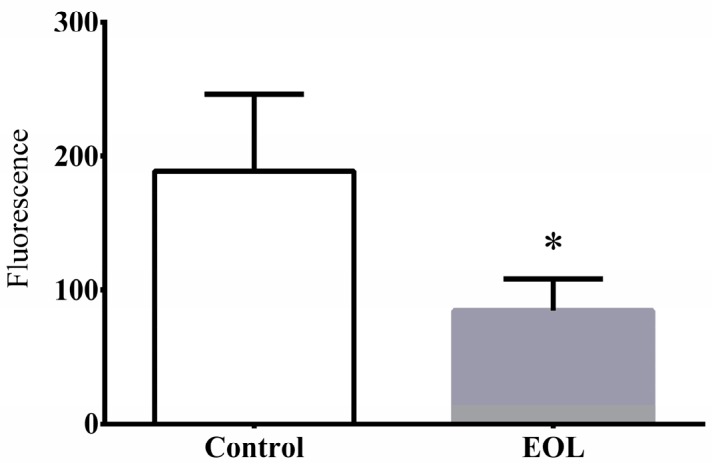
The effect of EOL on reducing ROS in *C. elegans*. Differences were considered to be significant at *p* < 0.05 (*).

Under the thermal condition, the ROS levels in the *C. elegans* rose sharply, producing more free radicals, which were the main reason of aging and destruction of cellular structure [39]. Some reports showed that the survival rate was lower with a high ROS level in *C. elegans*. However, extracts from natural plant could reduce the increasing ROS levels. José et al. found extracts from the Zalema grape pomace could increase the survival rate of worms and reduce the ROS level [35]. A similar outcome was obtained by Liao, using extracts from curcumin [19]. Hence, to investigate whether the EOL could reduce the ROS levels, worms were treated with EOL sample. Compared to the control group, the ROS level was significantly decreased by 54.15% (Figure 5). Therefore, the EOL might increase resistance to thermal stress by reducing the increasing ROS in *C. elegans*.

#### 2.3.5. The Expression of HSP-16.2

**Figure 6:**
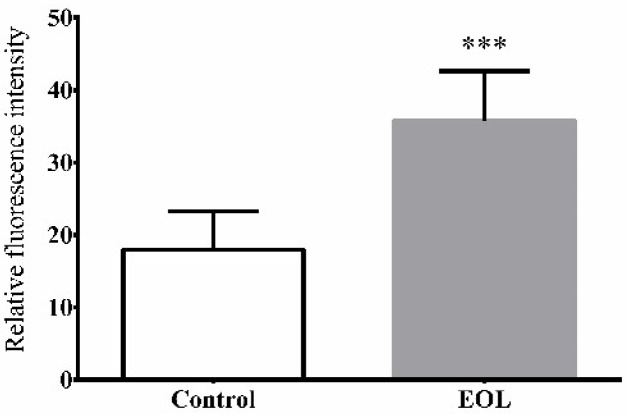
The effect of EOL on expression of HSP-16.2 in *C. elegans*. Differences were considered significant at *p* < 0.001 (***).

**Figure 7:**
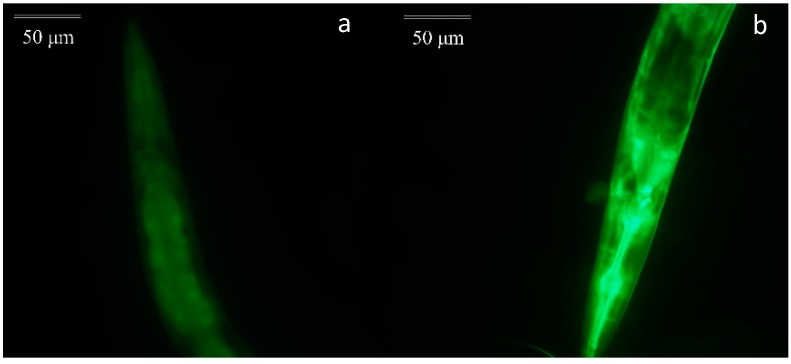
The expression of HSP-16.2 in *C. elegans*. (**a**) Treated with 1% DMSO. (**b**) Treated with EOL.

When an organism is placed in harsh environments like thermal stress, heat shock proteins (HSPs) are produced to prevent protein interaction which has a bad effect on the health of cells. Thus, every cell would maintain normal physiological activities with the help of HSPs. Moreover, HSPs have an influence on the resistance of *C. elegans* to stress [40]. Therefore, to value the affection of EOL on the expression of HSP-16.2 in *C. elegans*, a test was conducted by using the transgenic strain. Compared to the control group, the expression of HSP-16.2 was increased (Figure 6). Therefore, EOL could enhance the expression of HSP-16.2, to improve the resistance of *C. elegans* to thermal stress (Figure 7). Patrícia found Guarana extract could reduce intracellular ROS and increased the expression HSP-16.2 [41]. Therefore, we could assume that the EOL could not only lower the ROS level but could also increase the expression of HSP-16.2, to protect the whole organism against severe conditions.

#### 2.3.6. The Antioxidant Enzymes Activities and MDA Levels

**Figure 8:**
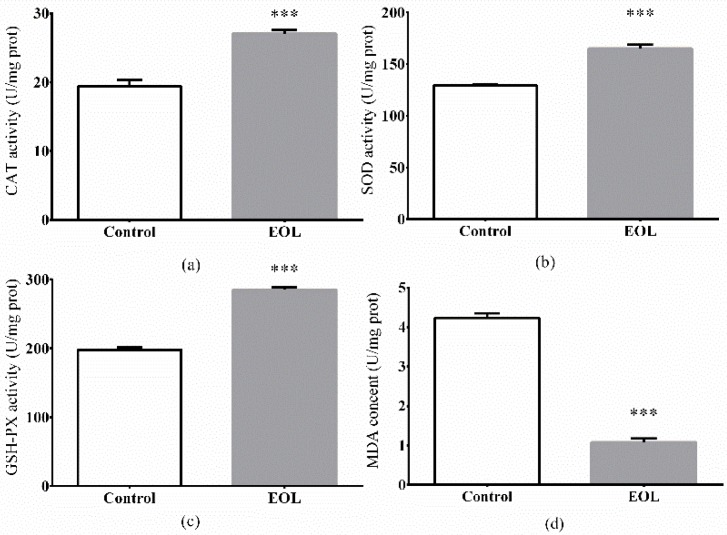
Effect of EOL on the activities of antioxidant enzymes under thermal stress. (**a**) CAT activity, (**b**) SOD activity, (**c**) GSH-PX activity, and (**d**) MDA content. Differences were considered to be significant at *p* < 0.001 (***).

ROS quickly accumulates in cells as a result of thermal stress, therefore, leading to oxidative damage of tissue, and consequently, accelerated death of organisms [42,43]. In vivo, antioxidant enzymes including CAT, SOD, GSH-Px play the main role in defending the organ system against over-production of ROS [44]. SOD has been documented to play a crucial role in turning free radicals into water and H_2_O_2_ [45]. CAT is regarded as the principal H_2_O_2_ scavenging enzyme. GSH-Px is also considered to be a part of the enzymatic scavenger systems to protect organisms from environmental stress [46]. After treatment with EOL, for 48 h, the CAT, GSH-Px, and SOD activities were found to have increased by 10.81%, 52.23%, and 30.97%, respectively, compared to the control group (Figure 8).

Polyunsaturated fatty acid components of phospholipids in cellular membranes, were sensitive to the free radical attack. Therefore, when ROS attacked the cellular membranes, MDA was produced by free radical-initiated oxidative damage of polyunsaturated fatty acids. The MDA was a consequence of lipid peroxidation possessing cytotoxicity [47]. In this study, the MDA content decreased by 72.96%, in comparison to the control group (Figure 8d).

Previous studies have found that the antioxidant enzymes promoted the protection of worms under harsh conditions, after *C. elegans* was treated with extracts from natural plants. Feng et al. found the polysaccharides from *P. notoginseng* could increase the activities of CAT and SOD [37]. Therefore, the hypothesis could be delivered that the EOL cleared the increasing free radicals produced by ROS by promoting the activities of inner antioxidant enzymes, such as CAT, SOD, and GSH-Px.

#### 2.3.7. Nuclear Translocation of *daf-16*::GFP

**Figure 9:**
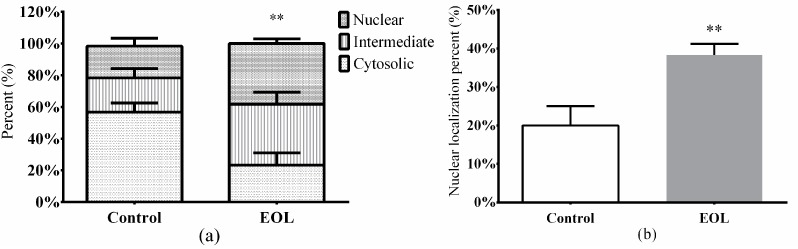
The effect of EOL on the transcriptional activity of *daf-16*. (**a**) The subcellular location of *daf-16*. (**b**) The *daf-16* nuclear localization percent. Differences were considered significant at *p* < 0.01 (**).

**Figure 10:**
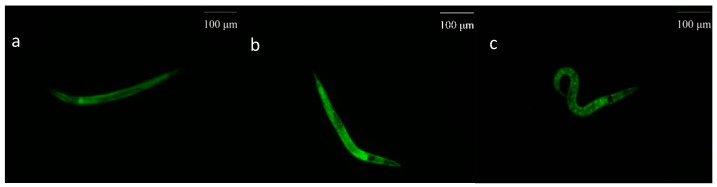
The transcriptional activity of *daf-16*. (**a**) The cytosolic localization of DAF-16::GFP. (**b**) Both cytosolic and nuclear localization of DAF-16::GFP. (**c**) The nuclear localization of DAF-16::GFP.

In *C. elegans*, the *daf-16* transcription factor could reinforce the resistance to stress via certain pathways [48]. With pretreatment of EOL for 24 h, the *daf-16* nuclear localization was significantly increased, by 18.33% (*p* < 0.001) (Figure 9 and Figure 10), the cytosolic localization was reduced to about 23.34% (*p* < 0.001) (Figure 9a). After the *daf-16* transcription factor turns to the nucleus, the expression of relevant genes would be activated, such as the genes of antioxidant enzymes. Studies have showed that extracts from plants could accelerate the *daf-16* nuclear localization. Extracts from Cistanche deserticola could well increase *daf-16* nuclear localization [49]. Feng et al. found that the polysaccharide extracts from *P. notoginseng* accelerated the *daf-16* translocation factor nuclear localization, while the activities of antioxidant enzymes, such as CAT and SOD, were enhanced [37,38]. Therefore, in this study, when treated with EOL, the *daf-16* nuclear localization had increased, which was associated with the increasing activities of antioxidant enzymes. Hence, the EOL might enhance the activities of the antioxidant enzymes via activation of the *daf-16* translocation factor.

## 3. Materials and Methods

### 3.1. Materials and Chemicals

Olive leaves of Pendolino were collected in July 2017 from the Olive planting base in Beihe, Xichang, Sichuan (China). The olive leaves were washed and dried at 45 °C. All dried samples were ground to fine powder screened by a 60 mesh sieve.

Nitrobluetetrazolium (NBT), phenazine methosulfate (PMS), 2,2-Diphenyl-1-picryl-hydrazyl (DPPH), and dihydronicotineamide adenine dinucleotide (NADH) were purchased from Sigma Chemical Co. (St. Louis, MO, USA). Methanol, ethanol, aluminum chloride, coomassie brilliant blue G-250, calcium chloride, magnesium sulfate, sulphuric acid, agar, cholesterol, yeast extract powder, peptone, and sodium chloride were purchased from the Chengdu Kolong Chemical Factory (Chengdu, China). All chemicals were analytical grade.

### 3.2. Methanol Extraction from Olive Leaves

Extraction of olive leaves was carried out according to the method stated in [50], with some modifications. Briefly, 20 g powder was mixed with 1 L methanol at 50 °C for 46 min, through ultrasound-assisted extraction with a power of 270 W by ultrasonic device (KQ300DV, 40 kHz, Kunshan Ultrasonic Instrument Co., Jiangsu, China). After being concentrated by a rotary evaporator, the concentration was dried in a vacuum drying oven, and then was kept at 4 °C.

### 3.3. Chemical Class Determination of EOL

#### 3.3.1. Polyphenols Content

Polyphenols of EOL was determined by the Folin–Ciocalteu reagent method [51]. Briefly, after being resolved in methanol, 0.1 mL EOL sample was added to 0.25 mL Folin–Ciocalteu reagent and 1 mL sodium carbonate solution (7.5% *w*/*v*), then the solution was diluted with distilled water, to 10 mL. The reaction mixture was incubated at room temperature, for 2 h, in dark. Absorbance of the solution was measured at 760 nm by microplate reader (Spectramax M2, USA). The total polyphenol was calculated with a standard curve made with the gallic acid standard as a reference.

#### 3.3.2. Flavonoids Content

Total flavonoids of EOL was determined by the aluminum chloride coloration method [52]. Briefly, 0.2 mL EOL sample was added to 0.4 mL aluminum chloride (1% *w*/*v*), then the solution was diluted with distilled water, to 2.5 mL. The reaction mixture was incubated at room temperature, for 10 min, in dark. Absorbance was measured at 415 nm. The total flavonoids was calculated with a standard curve made with the rutoside standard as a reference.

#### 3.3.3. Soluble Proteins Content

Soluble proteins of EOL was determined using the Coomassie Brilliant Blue (CBB) method with some slight modifications [53]. Ten grams of CBBG-250 was mixed with 5 mL 95% ethanol and 10 mL 85% phosphoric acid, then the mixture was diluted to 100 mL to obtain the CBBG-250 color reagent. About 0.1 mL of the EOL sample diluent was added to 0.5 mL CBBG-250 color reagent, then the solution was diluted to 1.5 mL. Absorbance of the reaction mixture was measured at 595 nm. The soluble proteins was calculated with a standard curve, with BSA as a reference.

#### 3.3.4. Soluble Sugars Content

Soluble sugars of EOL was determined by the phenol–sulfuric acid method [54]. Briefly, 0.1 mL EOL sample was mixed with 0.1 mL 6% phenol solution and 0.5 mL sulphuric acid, then absorbance was measured at 490 nm. The soluble sugars was calculated with a standard curve made with glucose as a reference.

#### 3.3.5. Free Amino Acids Content

Free amino acids of EOL was determined by the ninhydrin colorimetry method with slight modifications [55]. Basically, 1 mL EOL sample was mixed with 0.5 mL 2% ninhydrin and 0.5 mL phosphate buffered saline (pH = 8.0), then the mixture was incubated at 99 °C for 15 min. The absorbance of the reaction solution was measured at 570 nm, after being diluted to 25 mL. Free amino acids was calculated with a standard curve made with glutamate standard as a reference.

### 3.4. In Vitro Antioxidant Activity Assays

#### 3.4.1. DPPH Radical Scavenging Assay

EOL was resolved in methanol to different concentrations. The DPPH radical scavenging abilities of EOL were determined by the method described in [56], with some slight modifications. In brief, 35 μL ascorbic acid (Vc)—used as positive control—and the EOL sample (0–1.2 mg/mL), were added to 165 μL of DPPH ethanol solution (0.4 mM), respectively. The reaction solutions were incubated at 37 °C, for 10 min, in the dark, and then absorbance was measured at 517 nm by microplate reader (Spectramax M2, USA). The DPPH radical scavenging ability was calculated by the following formula:

DPPH radical scavenging activity (%) = (1 − A_1_/A_0_) × 100 (1)

where A_1_ is the absorbance of the reaction solution with the sample and A_0_ is the control group replaced with distilled water.

#### 3.4.2. Superoxide Radical Scavenging Assay

The superoxide radical scavenging ability of the EOL was determined by the method described in [57], with some slight modifications. In bried, 0.2 mL Vc—used as a positive control—and the EOL sample (0–1.2 mg/mL), were mixed with 0.2 mL NBT (0.08 mM), 0.4 mL NADH (0.25 mM), and 0.2 mL PMS (0.06 mM), respectively. Then the reaction solution was measured at 560 nm after being incubated at room temperature, for 10 min, in dark. The superoxide radical scavenging ability was calculated by the following formula:

Superoxide radical scavenging ability (%) = (1 − A_1_/A_0_) × 100 (2)

where A_1_ is the absorbance of the reaction solution with the sample and A_0_ is the control group replaced with distilled water.

#### 3.4.3. Reducing Power Assay

The reducing power of EOL was determined by the method described in [58], with some slight modifications. About 0.2 mL of the EOL sample (0–1.2 mg/mL) was added to 0.5 mL phosphate buffer (0.2 M) and 0.5 mL K3[Fe(CN_6_)] (1% *w*/*v*). The mixed solution was incubated at 50 °C, for 20 min, and subsequently cooled at 0 °C, for 5 min, after adding 0.5 mL TCA (10% *w*/*v*). About 0.5 mL aliquot of supernatant fluid was added to 0.5 mL distilled water and 0.1 mL FeCI_3_ (0.1% *w*/*v*). Then, the absorbance of the reaction solution was measured at 700 nm, after being incubated at room temperature, for 10 min. Increasing absorbance was directly associated with an increasing reducing power.

### 3.5. In Vivo Antioxidant Activity Assays

#### 3.5.1. *C. elegans* and Culture Conditions

Wild-type N2, TJ375 (gpls[*hsp-16.2*::GFP]), TJ356 *daf-16*::gfp (zIs356 (pDAF-16::DAF-16-GFP; rol-6)). *C. elegans* and *Escherichia coli* OP50 strain were obtained from the Caenorhabditis Genetics Center (CGC). The *C. elegans* were maintained on nematode growth medium (NGM) agar plates, with a layer of *E. coli* OP50 as food sources, at 20 °C. Synchronized worms were obtained by the sodium hypochlorite method [59].

#### 3.5.2. Food Clearance Assay

The optimal concentration of the EOL sample was determined by a food clearance assay, described in previous study [60]. Approximately 30 synchronized L4 nematodes grew in an S medium, containing EOL and OP50 in 96-well plates at 20 °C. The absorbance of the S medium was measured at 600 nm for 6 days.

#### 3.5.3. Fertility Assay

Synchronized L4 larva worms were transferred to a fresh NGM, coated with 0.4 mg/mL EOL sample or 1% DMSO, and were subsequently trained for 48 h. Four worms were transferred to a new NGM incubated for 1.5 h, then the number of eggs that had incubated to L2 or L3 stage, were counted [61]. All experiments were conducted for three times, individually.

#### 3.5.4. Thermal Stress Assay

The thermal stress assay was carried out according to the method with some slight modifications [62]. Briefly, after the sodium hypochlorite treatment, the eggs were transferred onto the NGM containing the *E. coli* OP50, until the L4 larvae stage. Subsequently, the L4 larva worms were transferred to a new fresh NGM, containing 0.4 mg/mL of the EOL sample and 25 μM 5-FUDR, for 48 h, while the control group was coated with 1% DMSO. 5-FUDR could prevent worms from reproduction. To determine the effect of EOL on the heat shock tolerance of the worms, the control and treated worms were incubated at 35 °C for 5 h, then scored for viability.

#### 3.5.5. Determination of the ROS Level

ROS level determination was conducted by the method described in [19]. Briefly, the synchronized L1 worms were transferred to a fresh NGM plate containing the EOL sample or 1% DMSO. After 24 h exposure, the plates were placed at 35 °C for 1 h. Then the worms were washed three times by M9, to remove the OP50. Subsequently, the worms were incubated in the M9-contained 50 μM DCFH-DA, for 0.5 h, then the fluorescence was measured by microplate reader (Spectramax M2, USA).

#### 3.5.6. Visualization of the HSP-16.2::GFP

Carrying a *hsp-16.2*::GFP, the TJ375 strain was used to test the expression of HSP-16.2. Briefly, synchronized L1 worms were transferred to a fresh NGM plate containing the EOL sample or 1% DMSO. After 24 h exposure, the worms were fixed on a slide with 15 mM sodium azide, after being placed at 35 °C for 1h. HSP-16.2::GFP fluorescence was examined by a fluorescence microscopy (Olympas-BX51TRF, Japan).

#### 3.5.7. Measurement of Antioxidant Enzymes and the MDA Level

To assess the SOD, CAT, and GSH-PX activities and the MDA level of *C. elegans*, under thermal stress, the control and treated worms were incubated at 35 °C for 3 h, then harvested by an M9 buffer. The worms were crushed by an ultrasonic wave to obtain a suspension. Then, the suspension was centrifuged at 2500 rpm/min, for 10 min. The supernatant liquid was determined for the protein content, the antioxidant enzyme activities, as well as the MDA level, according to the instructions of the assay kits (Nanjing Jiancheng Bioengineering Institute, Nanjing, China).

#### 3.5.8. Nuclear Localization of *daf-16*

Carrying a *daf-16*::GFP, the TJ356 stain was used to test the translization of *daf-16*, in the nucleus. Briefly, synchronized L1 worms were transferred to a fresh NGM plate, with the absence or presence of the EOL sample. After 24 h treatment, the worms were fixed on a microscopy slide with 15 mM sodium azide, then the subcellular DAF-distribution was observed under a fluorescence microscope (Olympas-BX51TRF, Japan). The location of *daf-16* was categorized as cytosolic, intermediate, and nuclear.

### 3.6. Statistical Analyses

The data were analyzed statistically by Student’s *t*-test (*t*-test) (GraphPad Prism 6) (GraphPad Software, Inc., La Jolla, CA, USA). The fluorescence intensity was quantified by the Image-J software (National Institutes of Health, Bethesda, MD, USA). All values were expressed as mean ± standard deviation (SD), *n* = 3. All experiments were performed in triplicates. Difference at the *p* < 0.05 level was considered to be significantly different.

## 4. Conclusions

In this study polyphenols was found to be the main compound in the EOL. In the investigations, it was obvious that the EOL expressed a good antioxidant ability. The most effectively scavenging ability of the DPPH radical was 91.03%, with an IC_50_ of 0.38 mg/mL. Under heat stress, the EOL could strength the resistance in the *C. elegans*, by conspicuously reducing the ROS level and increasing the expression of the HSP-16.2. The daf-16 transcription factor turned into the nucleus, and then activated the downstream target genes. Hence, the activities of antioxidant enzymes, such as SOD, CAT, and GSH-P_X_, were enhanced and the MDA content was sharply lowered. This study provided a definite evidence that extracts from olive leaves could efficiently scavenge free radicals in vitro and significantly increase expression of antioxidant enzymes in *C. elegans*, under acute stress. Therefore, olive leaves could be a good choice when it comes to antioxidants. The results in this study also laid a theoretical foundation for further development and utilization of olive leaves, which possess a potentially economic as well as medical values.

## Figures and Tables

**Table 1:** The chemical class of extracts from olive leaves (EOL).

**Compounds**	**Standard Caves**	**Contents**
Polyphenols	y = 1.6166x + 0.0147	41.77 ± 2.38%
Flavonoids	y = 0.1793x − 0.0012	30.01 ± 0.76%
Soluble proteins	y = 1.0645x + 0.0296	20.40 ± 0.69%
Soluble sugars	y = 5.7114x + 0.0904	14.14 ± 0.29%
Free amino acids	y = 1.6031x − 0.1964	0.09 ± 0.02%

## References

[1] Ying J., Liang G. (2012). Nutritive composition and health care function of olive oil. Acad. Period. Farm Prod. Prcess..

[2] Papadopoulos G., Boskou D. (1991). Antioxidant effect of natural phenols on olive oil. J. Am. Oil Chem. Soc..

[3] Hull W.E. (2000). Olive-oil consumption and health: The possible role of antioxidants. Lancet Oncol..

[4] Owen R.W., Giacosa A., Hull W.E., Haubner R., Spiegelhalder B., Bartsch H. (2000). The antioxidant/anticancer potential of phenolic compounds isolated from olive oil. Eur. J. Cancer.

[5] El S.N., Karakaya S. (2009). Olive tree (*Olea europaea*) leaves: Potential beneficial effects on human health. Nutr. Rev..

[6] María D.P., Feliciano P.C., María D.L.C. (2017). Selective ultrasound-enhanced enzymatic hydrolysis of oleuropein to its aglycon in olive (*Olea europaea* L.) leaf extracts. Food Chem..

[7] Caixia G., Chengzhang W., Chengying J. (2006). Chemical composition and processing utilization of olive. For. Sci. Technol. Dev..

[8] Jianzhong Y., Chengzhang W., Hongxia C., Hao Z. (2011). Variation Rule of Hydroxytyrosol Content in Olive Leaves. Chem. Ind. For. Prod..

[9] Rao F., Yuting Z., Yiran G., Fengxai L., Fang C. (2014). Determination of phenolic contents and antioxidant activities of extracts of *Jatropha curcas* L. seed shell, a by-product, a new source of natural antioxidant. Ind. Crop. Prod..

[10] Aruoma O.I. (1998). Free radicals, oxidative stress, and antioxidants in human health and disease. J. Am. Oil Chem. Soc..

[11] Maia T., Phillip F.P., Debebe G., Jayashree N., David R.H. (2006). Reactive oxygen species cerebral autoregulation in health and disease. Pediatr. Clin. N. Am..

[12] Harman D. (1972). The Biologic Clock: The Mitochondria?. J. Am. Geriatr. Soc..

[13] Simon H.U., Haj-Yehia A., Levi-Schaffer F. (2000). Role of reactive oxygen species (ROS) in apoptosis induction. Apoptosis.

[14] Valko M., Leibfritz D., Moncol J., Mark T.D., Milan M., Joshua T. (2007). Free radicals and antioxidants in normal physiological functions and human disease. Int. J. Biochem. Cell B.

[15] Visioli F., Poli A., Gall C. (2002). Antioxidant and other biological activities of phenols from olives and olive oil. Med. Res. Rev..

[16] Simona D.M., Carmen F., Franco Z., Antonella N., Lina A., Gennaro R., Maria I. (2014). Antioxidant activity and chemical components as potential anticancer agents in the olive leaf (*Olea europaea* L. cv Leccino.) Decoction. Anti-Cancer Agent Med. Chem..

[17] Shen P., Yue Y., Zheng J., Park Y. (2017). *Caenorhabditis elegans: A* convenient in vivo model for assessing the impact of food bioactive components on obesity, aging, and alzheimer’s disease. Annu. Rev. Food Sci. Technol..

[18] Lithgow G.J., Walker G.A. (2002). Stress resistance as a determinate of *C. elegans* lifespan. Mech. Ageing Dev..

[19] Liao H.C., Yu C.W., Chu Y.J., Li W.H., Hsieh Y.C., Wang T.T. (2011). Curcumin-mediated lifespan extension in *Caenorhabditis elegans*. Mech. Ageing Dev..

[20] Yanhong L., Bo J., Tao Z., Wanmeng M., Jian L. (2007). Antioxidant and free radical-scavenging activities of chickpea protein hydrolysate (CPH). Food Chem..

[21] Zhang Y., Mi D.-Y., Wang J., Luo Y.-P., Yang X., Dong S., Ma X.-M., Dong Z. (2018). Constituent and effects of polysaccharides isolated from Sophora moorcroftiana, seeds on lifespan, reproduction, stress resistance, and antimicrobial capacity in *Caenorhabditis elegans*. Chin. J. Nat. Med..

[22] Ishikado A., Sono Y., Matsumoto M., Stacey R.S., Aya O., Masashi G., George L.K., Blackwell T.K., Taketoshi M. (2013). Willow bark extract increases antioxidant enzymes and reduces oxidative stress through activation of Nrf2 in vascular endothelial cells and *Caenorhabditis elegans*. Free Radical Biol. Med..

[23] Cañuelo A., López B.G., Liñán P.P., Lara E.M., Siles E., Vizuete A.M. (2012). Tyrosol, a main phenol present in extra virgin olive oil, increases lifespan and stress resistance in *Caenorhabditis elegans*. Mech. Ageing Dev..

[24] Le F.F., Tena M.T., Ríos A., Valcarcel M. (1998). Supercritical fluid extraction of phenol compounds from olive leaves. Talanta.

[25] Prabhakar K.R., Veeresh V.P., Vipan K., Sudheer M., Priyadarsini K.I., Satish R.B.S.S., Unnikrishnan M.K. (2006). Bioactivity-guided fractionation of Coronopus didymus: A free radical scavenging perspective. Phytomedicine.

[26] González C.S. (2002). Methods used to evaluate the free radical sacavening activity in foods and biological systems: Review. Food Sci. Technol. Int..

[27] Brahmi F., Mechri B., Dabbou S., Dhibi M., Hammami M. (2012). The efficacy of phenolics compounds with different polarities as antioxidants from olive leaves depending on seasonal variations. Ind. Crop. Prod..

[28] Abaza L., Youssef B.N., Djebali H.M., Faouzia H., Methenmi K. (2011). Chétoui olive leaf extracts: Influence of the solvent type on phenolics and antioxidant activities. Grasas Y Aceites.

[29] Siddhuraju P., Klaus B. (2003). Antioxidant Properties of Various Solvent Extracts of Total Phenolic Constituents from Three Different Agroclimatic Origins of Drumstick Tree (*Moringa oleifera* Lam.) Leaves. J. Agric. Food Chem..

[30] Yen G.C., Duh P.D., Tsai C.L. (1993). Relationship between antioxidant activity and maturity of peanut hulls. J. Agric. Food Chem..

[31] Falleh H., Ksouri R., Chaieb K., Bouraoui N.K., Trabelsi N., Boulaaba M., Abdelly C. (2008). Phenolic composition of *Cynara cardunculus*, L. organs, and their biological activities. CR Biol..

[32] Xian X., Cao J., Zheng Y., Quanxi W., Jianbo X. (2014). Flavonoid concentrations and bioactivity of flavonoid extracts from 19 species of ferns from China. Ind. Crop. Prod..

[33] Nadia D., Nicola M., Daniela R., Immacolata F., Nunziatina D.T., Souad A., Lorella S., Luigi M. (2015). Phenolic compounds from *Olea europaea* L. possess antioxidant activity and inhibit carbohydrate metabolizing enzymes in vitro. Evid. Based Complement Alternat. Med..

[34] Vayndorf E.M., Lee S.S., Liu R.H. (2013). Whole apple extracts increase lifespan, healthspan and resistance to stress in *Caenorhabditis elegans*. J. Funct. Foods.

[35] Jara P.M., González M.S., Escudero G.M., Hernanz D., Dueñas M., González P.A., Heredia F., Santos B.C. (2013). Study of Zalema Grape Pomace: Phenolic Composition and Biological Effects in *Caenorhabditis elegans*. J. Agric. Food Chem..

[36] Chattopadhyay D., Thirumurugan K. (2018). Longevity promoting efficacies of different plant extracts in lower model organisms. Mech. Ageing Dev..

[37] Shiling F., Haoran C., Zhou X., Shian S., Ming Y., Jing L., Chunbang D. (2015). Thermal stress resistance and aging effects of *Panax notoginseng*, polysaccharides on *Caenorhabditis elegans*. Int. J. Biol. Macromol..

[38] Shiling F., Haoran C., Zhou X., Ming Y., Yan H., Jinqiu L., Ruiwu Y., Lijun Z., Chunbang D. (2018). *Panax notoginseng*, polysaccharide increases stress resistance and extends lifespan in *Caenorhabditis elegans*. J. Funct. Foods.

[39] Wulf D. (2002). Free Radicals in the Physiological Control of Cell Function. Physiol. Rev..

[40] Hsu A.L. (2003). Regulation of Aging and Age-Related Disease by DAF-16 and Heat-Shock Factor. Science.

[41] Patrícia F.B., Giovanna M.M.S., Franciny A.P., Rodrigo M.C., Cecília V.N., Riva P.O. (2018). Guarana (*Paullinia cupana*) Extract Protects *Caenorhabditis elegans* Models for Alzheimer Disease and Huntington Disease through Activation of Antioxidant and Protein Degradation Pathways. Oxid. Med. Cell. Longev..

[42] Naoaki I., Nanami S.M., Kohichiro M., Kayo Y., Takamasa I., Philip S.H., Satoru F. (2004). Coenzyme Q10 can prolong *C. elegans* lifespan by lowering oxidative stress. Mech. Ageing Dev..

[43] Zhuanhua W., Xiaoli M., Jiao L., Xiaodong C. (2016). Peptides from sesame cake extend healthspan of *Caenorhabditis elegans*, via upregulation of skn-1, and inhibition of intracellular ROS levels. Exp. Gerontol..

[44] Beckman K.B., Ames B.N. (1998). The free radical theory of aging matures. Physiol. Rev..

[45] Celino F.T., Yamaguchi S., Miura C., Ohta T., Tozawa Y., Iwai T., Miura T. (2012). Tolerance of spermatogonia to oxidative stress is due to high levels of Zn and Cu/Zn superoxide dismutase. PLoS ONE.

[46] Xia X.F., Zheng J.J., Shao G.M., Wang J.L., Liu X.S., Wang Y.F. (2013). Cloning and functional analysis of glutathione peroxidase gene in red swamp crayfish Procambarus clarkii. Fish Shellfish Immunol..

[47] Bokov A., Chaudhuri A., Richardson A. (2004). The role of oxidative damage and stress in aging. Mech. Ageing Dev..

[48] Ogg S., Paradis S., Gottlieb S., Patterson G.I., Lee L., Tissenbaum H.A., Ruvkun G. (1997). The Fork head transcription factor DAF-16 transduces insulin-like metabolic and longevity signals in *C. elegans*. Nature.

[49] Wei C., Hongru L., Congmin W., Xiaohua L., Menglu S., Zhenzhou Y., Xinyan C., Hongbing W. (2017). Echinacoside, a phenylethanoid glycoside from Cistanche deserticola, extends lifespan of *Caenorhabditis elegans* and protects from Aβ-induced toxicity. Biogerontology.

[50] Wang B., Qu J., Luo S., Feng S., Li T., Yuan M., Huang Y., Liao J., Yang R., Ding C. (2018). Optimization of ultrasound-assisted extraction of flavonoids from olive (*Olea europaea* L.) leaves, and evaluation of their antioxidant and anticancer Activities. Molecules.

[51] Popova M., Bankova V., Butovska D., Petkov V., Damyanova B.N., Sabatini A.G., Marcazzan G.L., Bogdanov S. (2004). Validated methods for the quantification of biologically active constituents of Poplar-type propolis. Phytochem. Anal..

[52] Meda A., Lamien C.E., Romito M., Millogo J., Nacoulma O.G. (2005). Determination of the total phenolic, flavonoid and proline contents in Burkina Fasan honey, as well as their radical scavenging activity. Food Chem..

[53] Grintzalis K., Georgiou C.D., Schneider Y.J. (2015). An accurate and sensitive Coomassie Brilliant Blue G-250-based assay for protein determination. Anal. Biochem..

[54] DuBois M., Gilles K.A., Hamilton J.K., Rebers P.A., Smith F. (1956). Colorimetric method for determination of sugars and related substances. Anal. Chem..

[55] Yemm E.W., Cocking E.C. (1955). The determination of amino-acids with ninhydrin. Analyst.

[56] Brand W.W.M., Cuvelier M.E., Berset C. (1995). Use of a free radical method to evaluate antioxidant activity. LWT Food Sci. Technol..

[57] Jia Z., Tang M., Wu J. (1999). The determination of flavonoid contents in mulberry and their scavenging effects on superoxide radicals. Food Chem..

[58] Moein M.R., Moein S., Ahmadizadeh S. (2008). Radical scavenging and reducing power of Salvia mirzayanii subfractions. Molecules.

[59] Portadelariva M., Fontrodona L., Villanueva A., Ceron J. (2012). Basic *Caenorhabditis elegans* methods: Synchronization and observation. J. Vis. Exp..

[60] Cindy V., Hemant V., Nicola W., Emily A.B., Brent R.S., Anne C.H. (2007). Identification of potential therapeutic drugs for huntington’s disease using *Caenorhabditis elegans*. PLoS ONE.

[61] Bany I.A., Dong M.Q., Koelle M.R. (2003). Genetic and cellular basis for acetylcholine inhibition of *Caenorhabditis elegans* egg-laying behavior. J. Neurosci..

[62] Mekheimer R.A., Sayed A.A., Ahmed E.A. (2012). Novel 1,2,4-triazolo[1,5-a]pyridines and their fused ring systems attenuate oxidative stress and prolong lifespan of *Caenorhabiditis elegans*. J. Med. Chem..

